# Genetic polymorphisms in *ABCB1* are correlated with the increased risk of atorvastatin-induced muscle side effects: a cross-sectional study

**DOI:** 10.1038/s41598-023-44792-2

**Published:** 2023-10-19

**Authors:** Ninoslava Lalatović, Maša Ždralević, Tanja Antunović, Snežana Pantović

**Affiliations:** 1 Faculty of Medicine, University of Montenegro, Kruševac bb, 81000 Podgorica, Montenegro; 2 Institute for Advanced Studies, University of Montenegro, Cetinjska 2, 81000 Podgorica, Montenegro; 3 Center for Clinical Laboratory Diagnostic, Clinical Center of Montenegro, Ljubljanska bb, 81000 Podgorica, Montenegro

**Keywords:** Molecular medicine, Risk factors

## Abstract

Genetic factors are recognized as risk factors for statin-associated muscle symptoms (SAMS), which are the most common cause of statin intolerance. The aim of this study was to determine whether there is an association between polymorphisms 1236C > T, 2677G > T/A, and 3435C > T in the *ABCB1* gene, encoding the efflux transporter of statins, and SAMS, as results on this topic are still controversial. A cross-sectional study was conducted on patients with or without SAMS using atorvastatin. The influence of non-genetic variables on SAMS was also evaluated. Our results show that patients with TT genotype in 1236C > T, 2677G > T/A, and 3435C > T polymorphisms had higher risk of developing SAMS, compared to wild type and heterozygous carriers together (OR 4.292 *p* = 0.0093, OR 5.897 *p* = 0.0023 and OR 3.547 *p* = 0.0122, respectively). Furthermore, TTT/TTT diplotype was also associated with a higher risk of SAMS, OR 9.234 (*p* = 0.0028). Only family history of cardiovascular disease was found to be a risk factor for SAMS, in addition to the known non-genetic variables. We believe that *ABCB1* genotyping has great potential to be incorporated into clinical practice to identify high-risk patients in a timely manner.

## Introduction

Statins are the drugs of first choice in the treatment of dyslipidemia^1^. However, some patients do not tolerate statin therapy because of side effects, most often due to myalgia, and are forced to change therapy, or use the drug at a lower dose, which may result in decreased efficacy of the drug^2–4^. Although the mechanisms underlying statin associated muscle symptoms (SAMS) are still not fully understood, it is clear that elevated serum statin concentrations increase the risk of their development^5^.

Variations in genes encoding transporter proteins or enzymes that metabolize statins, may alter the pharmacokinetics of statin^6^. One of the most studied genes involved in the disposition of statins is the ATP-binding cassette subfamily member 1 (*ABCB1*), encoding the efflux protein transporter P-glycoprotein (P-gp), which is involved in the process of absorption as well as excretion of various types of drugs, and determines the concentration of the drug that reaches the target tissue^7^. The single nucleotide polymorphisms (SNPs) rs1128503 (1236C > T), rs2032582 (2677G > T/A), and rs1045642 (3435C > T) in this gene have been the most common subjects of investigation^8^. Although numerous pharmacokinetic studies have confirmed that these *ABCB1* polymorphisms result in higher area under the curve (AUC) of different statin types, findings on the association of these polymorphisms with SAMS are still controversial^9^. For example, one study reported that systemic exposure to atorvastatin was 55% higher and its elimination half-life 24% longer in subjects with the *ABCB1* TTT/TTT diplotype, compared with CGC/CGC^10^. Thus, it could be concluded that patients with the TT genotype will have a higher risk of SAMS, which some studies confirmed^11,12^, some refuted^13^, while others found no association^14–16^. The Clinical Pharmacogenetic Implementation Consortium (CPIC) has issued guidelines for the use of statins depending on the genotype of Solute Carrier Organic Anion Transporter Family Member 1B1 (*SLCO1B1*), ATP-Binding Cassette Subfamily G Member (*ABCG2*), and Cytochrome P450 Family 2 Subfamily C Member 9 (*CYP2C9*) genes with the aim to improve overall safety and adherence, and thus efficacy of therapy^17^. However, because of the lack of strong evidence in the literature, there are no guidelines for the use of statins depending on the *ABCB1* genotype.

Considering the importance of P-gp in the efflux of statins, the fact that variations in this gene could significantly affect the systemic concentration of the drug, and conflicting results of candidate gene association studies with SAMS, our goal was to determine whether there is an association between polymorphisms 1236C > T, 2677G > T/A, and 3435C > T in the *ABCB1* gene and the side effects of atorvastatin, the most commonly prescribed statin, at the muscle level. Our hypothesis was that the carriers of TT genotype would be associated with an increased risk of SAMS. It is hoped that this study will ultimately aid clinicians in guiding individual treatments of patients with dyslipidemia.

## Results

### Patient characterization

Out of total 110 participants, 41 subjects (37.27%) belonged to the AAMS group, whereas 69 (62.73%) were in the N-AAMS group. Clinical and demographic characteristics of these groups are shown in the Table 1.

**Table 1:** Clinical and demographic characteristics of AAMS group and N-AAMS group.

Variable	AAMS group (n = 41) Mean ± SD or number (%)	N-AAMS group (n = 69) Mean ± SD or number (%)	*p* value
Sex			
Male	27 (65.85)	41 (59.42)	0.5475
Female	14 (34.15)	28 (40.58)	
Age	60.80 ± 9.22	63.17 ± 8.38	0.3313
Hypertension			
Yes	28 (68.29)	55 (79.71)	0.2516
No	13 (31.71)	14 (20.29)	
Diabetes			
Yes	9 (21.95)	23 (33.33)	0.2781
No	32 (78.05)	46 (66.67)	
Angina pectoris			
Yes	9 (21.95)	22 (31.88)	0.2835
No	32 (78.05)	47 (68.12)	
CCI	2.71 ± 1.25	3.03 ± 1.38	0.2609
Family history of CVD			
Yes	33 (80.49)	39 (56.52)	0.0129*
No	8 (19.51)	30 (43.48)	
BMI	28.25 ± 3.25	28.27 ± 5.1	0.4676
History of smoking			
Yes	26 (63.41)	50 (72.46)	0.3944
No	15 (36.59)	19 (27.54)	
Alcohol consumption			
Yes	14 (34.15)	16 (23.19)	0.2691
No	27 (65.85)	53 (76.81)	
Physical activity per day			
< 30 min	23 (56.10)	33 (47.83)	0.4354
≥ 30 min	18 (43.90)	36 (52.17)	
Atorvastatin			
10 mg	6 (14.63)	11 (15.94)	0.6745
20 mg	25 (60.98)	46 (66.67)	
40 mg	10 (24.39)	12 (17.39)	
Duration of therapy in months	31.01 ± 36.78	28.33 ± 29.06	0.9212
CK (IU/L)	193 ± 248	90.32 ± 38.09	< 0.0001*

*AAMS* atorvastatin-associated muscle symptoms, *N-AAMS* non-atorvastatin-associated muscle symptoms, *CCI* Charlson Comorbidity Index, *CVD* Cardiovascular disease, *BMI* Body Mass Index, *CK* Creatine Kinase, *SD* Standard Deviation.

*Significant values are in bold.

There were no significant differences between the groups in terms of clinical and demographic variables, except for family history of CVD. Namely, the frequency of patients from AAMS group that had positive history of CVD was significantly higher, with respect to the N-AAMS.

### Distribution of alleles and genotypes of studied *ABCB1* polymorphisms and their correlation with statin-associated muscle symptoms

In order to assess the potential contribution of three selected *ABCB1* polymorphisms to SAMS related to the use of atorvastatin, we have first analyzed the frequencies of alleles, genotypes, haplotypes, and diplotypes in the total population (Table 2). The distribution of genotypes of all SNPs was consistent with Hardy–Weinberg equilibrium (*p* > 0.05).

**Table 2:** The frequencies of *ABCB1* alleles, genotypes, haplotypes and diplotypes in the study population (n = 110).

SNP ID	Allele	Allele frequency (%)	Genotype	Frequency of genotypes (%)	*p*-value	Haplotype	Frequency of haplotypes (%)	Diplotype	Frequency of diplotypes (%)
rs1128503	C	58.64	CC	35.45		TTT	35	TTT/TTT	11.82
	T	41.36	CT	46.36	0.898	TTC	2.73	TTC/TTT	3.64
			TT	18.18		CTT	2.27	CTT/TTT	0.91
rs2032582	G	56.82	GG	30.91		TGT	0.45	CGT/TTT	4.55
	T	40.45	GT	47.27		CTC	0.45	CGC/TTT	34.55
	A	2.73	GA	4.55	0.8803	CGC	45.91	TGC/TTT	2.73
			TT	16.36		TGC	2.73	CGC/TGT	0.91
			AA	0		TAT	0.45	CGC/TGC	2.73
			TA	0.91		CAC	1.36	CGC/TTC	1.82
rs1045642	C	53.18	CC	27.27		CGT	7.73	CTC/TAT	0.91
	T	46.82	CT	51.82	0.9136	CAT	0.91	CGC/CGC	20
			TT	20.91				CGC/CAC	2.73
								CGC/CGT	7.27
								CGT/CTT	2.73
								CGC/CTT	0.91
								CGT/CAT	0.91
								CGC/CAT	0.91

Next, we have analyzed allele, genotype and genetic model (dominant/recessive) frequencies of all three SNPs in AAMS and N-AAMS groups. The results are summarized in Table 3, and all three SNPs were shown to be correlated with SAMS.

**Table 3:** Association analysis of single nucleotide polymorphisms from *ABCB1* gene with atorvastatin associated muscle symptoms.

SNP ID	Genetic model	AAMS group number (%)	N-AAMS group number (%)	*OR*	95% CI	*p-*value	*p-*value threshold
rs1128503 (1236C>T)	T vs. C^a^	41 (50.00)/41 (50.00)^a^	50 (36.23)/88 (63.77)^a^	1.76^a^	1.006-3.108^a^	0.0488*^a^	0.05^a^
	TT vs. CT vs. CC^b^	12 (29.27)/17 (41.46)/12 (29.27)^b^	8 (11.59)/34 (49.28)/27 (39.13)^b^	–^b^	–^b^	0.0651^b^	0.025^b^
	TT + CT vs. CC^c^	29 (70.73)/12 (29.27)^c^	42 (60.87)/27 (39.13)^c^	1.554^c^	0.6634-3.43^c^	0.3124^c^	0.05^c^
	TT vs. CT + CC^d^	12 (29.27)/29 (70.73)^d^	8 (11.59)/61 (88.41)^d^	3.155^d^	1.144-8.745^d^	0.0386^d^	0.0167^d^
rs2032582 (2677G>T/A)	T (A) vs. G^a^	41 (50.00)/41 (50.00)^a^	54 (39.13)/84 (60.87)^a^	1.556^a^	0.894-2.725^a^	0.1237^a^	0.05^a^
	T(A)/T(A) vs. GT(A) vs. GG^b^	13 (31.71)/15 (36.58)/13 (31.71)^b^	6 (8.70)/42 (60.87)/21 (30.43)^b^	–^b^	–^b^	0.0045*^b^	0.025^b^
	T(A)/T(A) + GT(A) vs. GG^c^	28 (68.29)/13 (31.71)^c^	48 (69.57)/21 (30.43)^c^	0.9423^c^	0.4251-2.251^c^	>0.9999^c^	0.05^c^
	T(A)/T(A) vs. GT(A) + GG^d^	13 (31.71)/28 (68.29)^d^	6 (8.70)/63 (91.30)^d^	4.875^d^	1.65-14.32^d^	0.0034*^d^	0.0167^d^
rs1045642 (3435C>T)	T vs. C^a^	43 (52.44)/39 (47.56)^a^	60 (43.48)/78 (56.52)^a^	1.433^a^	0.8296-2.499^a^	0.2111^a^	0.05^a^
	TT vs. CT vs. CC^b^	14 (34.15)/15 (36.58)/12 (29.27)^b^	9 (13.04)/42 (60.87)/18 (26.09)^b^	–^b^	–^b^	0.0143*^b^	0.025^b^
	TT + CT vs. CC^c^	29 (70.73)/12 (29.27)^c^	51 (73.91)/18 (26.09)^c^	0.8529^c^	0.364-1.929^c^	0.8253^c^	0.05^c^
	TT vs. CT + CC^d^	14 (34.15)/27 (65.85)^d^	9 (13.04)/60 (86.96)^d^	3.457^d^	1.384-8.484^d^	0.0142*^d^	0.0167^d^

The genetic model is presented as follows: ^a^ Allele model, ^b^ Codominant model, ^c^ Dominant model; ^d^ Recessive model. *SNP* single nucleotide polymorphism, *AAMS* atorvastatin-associated muscle symptoms, *N-AAMS* non-atorvastatin-associated muscle symptoms, *OR* odds ratio.

*Significant values are in bold.

More precisely, the results showed that T allele in rs1128503 (1236C > T) polymorphism could be associated with SAMS, compared to the C allele, OR 1.76 (1.006–3.108, 95% CI, *p* = 0.0488). Interestingly, the frequency of the C and T alleles was the same in the AAMS group, but the T allele was significantly less frequent within the N-AAMS group. The statistical significance in recessive genetic model was lost after the post hoc test.

In the triallelic SNP rs2032582 (2677G >T/A), there was a statistically significant difference in the distribution between the genotypes. The frequency of the variant homozygous (T(A)/T(A)) was 3.64 times higher in the AAMS group compared to the N-AAMS group, and 3.5 and 7 times lower in the N-AAMS group compared to the wild type (GG) and the heterozygous, respectively (*p* = 0.0045). Carriers of both copies of variant allele T(A) were found to have higher risk of muscular SE, compared to wild type (GG) and carriers of only one variant allele GT(A) together, OR 4.875 (1.65–14.32, 95% CI, *p* = 0.0034).

When it comes to rs1045642 SNP (3435C > T), no significant difference was found in the distribution of T and C alleles between the AAMS and N-AAMS groups. However, there was a significant difference between genotypes, with the frequency of the TT genotype being 2.6-fold higher in the AAMS group compared with the N-AAMS group. Also, TT genotype within the N-AAMS group was twofold and 4.7-fold lower compared with the CC and CT genotypes, respectively (*p* = 0.0143). The statistical significance in the recessive genetic model (TT vs. CC + CT) OR 3.457 (1.384–8.484, 95% CI, *p* = 0.0142) between AAMS and N-AAMS groups, is consistent with the previous findings, that there is a higher probability of manifestation of adverse effects in the homozygous variant (TT) carriers.

Since 1236C > T, 2677G > T/A and 3435C > T *ABCB1* SNPs are in high linkage disequilibrium (LD)^8^, a correlation analysis between haplotypes, diplotypes and SAMS was also performed. The distribution of haplotypes and diplotypes among the AAMS and N-AAMS groups are given in the Table 4. In order to improve statistical power, only those haplotypes and diplotypes with frequencies greater than 1% were included in the analysis. The comparison of haplotypes/diplotypes between the groups was performed with respect to the haplotype/diplotype that had the highest frequency in the study population, which are the CGC haplotype, and CGC/TTT diplotype, respectively.

**Table 4:** Association analysis between haplotypes and diplotypes of SNPs from *ABCB1* gene and atorvastatin associated muscle symptoms.

Haplotype diplotype	AAMS group number (%)	N-AAMS group number (%)	OR (95% CI)	*p* value
CGC	35 (42.68)	66 (47.83)	–	–
TTT	37 (45.12)	40 (28.99)	1.744 (0.9402–3.123)	0.09
TTC	2 (2.44)	4 (2.9)	0.9429 (0.1728–4.217)	> 0.9999
CTT	2 (2.44)	3 (2.17)	1.257 (0.2148–6.377)	> 0.9999
TGC	2 (2.44)	4 (2.9)	0.9429 (0.1728–4.217)	> 0.9999
CGT	4 (4.88)	13 (9.42)	0.5802 (0.1955–1.731)	0.4194
CGC/TTT	12 (29.27)	26 (37.68)	–	–
TTT/TTT	10 (24.39)	3 (4.35)	7.222 (1.772–26.80)	0.0081*
TTC/TTT	2 (4.88)	2 (2.9)	2.167 (0.3050–14.80)	0.5902
CGT/TTT	2 (4.88)	3 (4.35)	1.444 (0.2308–7.773)	> 0.9999
CGC/TGC	2 (4.88)	1 (1.45)	4.333 (0.4515–64.75)	0.2646
CGC/CGC	10 (24.39)	12 (17.39)	1.806 (0.6091–5.392)	0.4048
CGC/CGT	1 (2.44)	7 (10.145)	0.3095 (0.0255–2.278)	0.4088
CGT/CTT	1 (2.44)	2 (2.9)	1.083 (0.0695–10.00)	> 0.9999

*AAMS* atorvastatin-associated muscle symptoms, *N-AAMS* non-atorvastatin-associated muscle symptoms, *OR* odds ratio.

*Significant values are in bold.

None of the haplotypes included in the analysis showed a statistically significant difference in distribution between the AAMS/N-AAMS groups. However, our analysis showed that the frequency of variant homozygous (TTT/TTT) of all three *ABCB1* SNPs was significantly higher (5.6 times higher) in the AAMS group than in the N-AAMS group, and that carriers of the TTT/TTT diplotype could be associated with a higher risk of SAMS, OR 7.222 (1.772–26.80, 95% CI, *p* = 0.0081).

### Correlation of non-genetic variables with SAMS

To investigate the possible influence of all the above-mentioned covariates (Table 1) on the development of SAMS, we performed a simple logistic regression analysis. Interestingly, neither dose nor duration of therapy was found to have an impact on the occurrence of SAMS; only family history of CVD was associated with the outcome. Participants with family history of CVD had a higher risk of developing SAMS (OR 3.173, 1.326–8.286, 95% CI, *p* = 0.0126) (data not shown). It should be emphasized that univariable logistic regression analysis yielded statistically significant results in the case of the recessive genetic model (TT vs CT + CC) for SNP 1236C > T OR 3.155 (1.178–8.868, 95% CI, *p* = 0.0240), which have lost statistical significance after the post hoc test (Table 3.) Then, we conducted multivariable logistic regression analysis for recessive genetic model of all three polymorphisms, as well as TTT/TTT diplotype, and included all environmental variables as covariates. Only results for genetic risk factors are presented in Table 5.

**Table 5:** Significant genetic risk factors for atorvastatin associated muscle symptoms adjusted for the non-genetic covariates.

Variable	OR crude (95% CI)^a^	*p* value^a^	OR adjusted (95% CI)^b^	*p* value^b^
TT vs. CT + CC 1236C > T	3.155 (1.178–8.868)	0.0240	4.292 (1.481–13.69)	0.0093
T(A)/T(A) vs. GT(A) + GG 2677G > T/A	5.943 (2.03–20.01)	0.0019	5.897 (1.975–20.06)	0.0023
TT vs. CT + CC 3435C > T	3.457 (1.352–9.25)	0.0107	3.547 (1.342–9.908)	0.0122
TTT/TTT vs. others	7.097 (2.009–33.33)	0.0047	9.234 (2.399–47.84)	0.0028

*OR* odds ratio.

^a^Univariable logistic regression.

^b^Multivariable logistic regression.

Multivariable logistic regression analysis showed that the associations of TT genotype of all three *ABCB1* polymorphisms, including TTT/TTT diplotype, on SAMS remained significant even after adjusting the model for all covariates. The same case was with the family history of CVD. It could be concluded that the influence of genotype and family history of CVD on the occurrence of SAMS are independent and both can be considered as risk factors.

## Discussion

The results of our study indicate that myalgia, the most common side effect in atorvastatin users, is associated with polymorphisms in the *ABCB1* gene. Indeed, we have shown that homozygous carriers of T allele in 1236C > T, 2677G > T/A, and 3435C > T SNPs have a higher risk of developing SAMS, compared with wild type and heterozygous carriers. The presence of a TT genotype in all three *ABCB1* polymorphisms increased the risk of SAMS almost twofold.

rs1128503 (1236C > T) is a synonymous SNP, which together with the rs1045642 (3435C > T) is thought to affect the process of co-translational protein folding^21^. Carriers of the TT genotype were shown to have significantly higher maximum plasma concentration (Cmax) and AUC of rosuvastatin than heterozygous carriers^22^. In addition, 1236 T carriers taking simvastatin showed significantly greater reduction in total cholesterol and LDL levels^23^, suggesting that they were correlated with the higher systemic concentration of the drug. This could subsequently lead to a higher risk of SAMS, as confirmed by our findings for atorvastatin (Table 3). Our results are consistent with the findings by Ferrari et al. regarding statistically significant difference in allele frequencies for 1236C > T polymorphism, but not for the dominant genetic model, in which they also found a statistically significant difference^12^, suggesting that the risk of SAMS increases with an increase in the number of copies of the T allele. Consistent with that, a significant difference in the distribution between homozygous and heterozygous carriers of the variant allele (TT vs CT) would be expected, but no statistically significant difference was observed (OR = 3, 0.8446–9.565, 95% CI, *p* = 0.1398)^12^. After the post hoc test, which accounted for multiple hypothesis testing adjustment, statistical significance was lost in the case of the recessive genetic model (Table 3), but both univariable and multivariable logistic regression analysis yielded statistically significant results for this genetic model (Table 5), which is consistent with findings by Ferrari et al.^12^.

The missense variant rs2032582 (2677G > T, A) is characterized by substitution of the amino acid alanine by serine or threonine^7^. As a consequence, several studies reported a significant decrease in *ABCB1* expression and/or a decrease in P-gp function in carriers of the variant allele^24^. This is supported by the fact that AUC and Cmax of rosuvastatin were significantly higher in non-G carriers compared with wild-type and heterozygous^22^. Moreover, a threefold greater decrease in LDL levels was observed in carriers of the T allele with respect to carriers of the G allele following atorvastatin administration^25^. Accordingly, non-G homozygous carriers were at higher risk of SAMS because of higher systemic exposure, which was confirmed by our study^26^.

rs1045642 (3435C > T) is a synonymous SNP known to lead to changes in drug pharmacokinetics, but the underlying mechanisms are still unknown. Most likely explanation is that the T allele affects the timing of co-translational folding and incorporation of P-gp into the cell membrane, resulting in a change of substrate binding site structure^7^. Accordingly, TT carriers can be expected to have an increase in systemic exposure of the substrate^27,28^, and therefore will be at higher risk for developing SAMS. Hoenig et al. reported that the frequency of the T allele was higher in the group of subjects with myalgia than in the control group^11^, which is in agreement with our results (Table 3), but this difference between alleles was not statistically significant in our case. This discrepancy could potentially be explained by the high frequency of heterozygous carriers (60.87%) in our control group. However, Hoenig et al. did not find a difference in distribution between genotypes or in the dominant genetic model (TT + TC vs CC, *p* > 0.9999)^11^, confirming that being a carrier of the T allele is not sufficient to be at risk for SAMS. The results of our study are in agreement with this, and furthermore, we showed that only the presence of the T allele on both homologous chromosomes can be considered as a risk factor for the development of SAMS. We have shown that there was a statistically significant difference in the distribution of genotypes of 3435C > T polymorphism, in agreement with Ferrari et al.^12^, but in their study no statistically significant difference was observed in the recessive genetic model (*p* = 0.090)^12^. A recent meta-analysis reported the lack of association between 3435C > T polymorphism and SAMS^29^, which is in contrast with our results, but it reported the effects of all statins together, which could account for the observed differences.

It is known that the TTT haplotype is associated with 80–100% lower P-gp activity compared with wild type^30^, and it is therefore expected that the risk for SAMS is higher in TTT carriers. Indeed, we found that the frequency of variant alleles for all 3 SNPs was higher in the AAMS compared to the N-AAMS group, although the difference was not statistically significant, except in the case of 1236C > T polymorphism (Table 3). These results are in contrast to the study by Fiegenbaum et al.^13^, which reported lower frequency of the T/T(A)/T haplotype in the group of patients with myalgia than in the control group^13^, while in our study the frequency of the TTT haplotype was 1.57-fold higher in the AAMS group compared with N-AAMS, although the difference was not statistically significant (Table 4). The study by Herman et al. reported no association of SAMS with *ABCB1* polymorphisms (1236C > T, 2677G > T, A 3435C > T) in patients on atorvastatin^15^. However, in the group of patients with myalgia, the AUC was 1.26 and 3.1 times higher for 2-hydroxy-atorvastatin and 4-hydroxy-atorvastatin respectively, compared to the control group^15^. On the other hand, in the study by Keskitalo et al., significantly higher systemic exposure of ortho- and para-hydroxy-atorvastatin was observed in carriers of the TTT/TTT diplotype compared with the wild type^10^. Elimination half-life of simvastatin, also a substrate for P-gp, was 30% higher in TTT/TTT carriers compared with CGC/CGC^31^. In our study, a higher frequency of the TTT/TTT diplotype was found in the group of patients with myalgia, which indicates that these patients are at higher risk for SAMS possibly because of longer retention and accumulation of active metabolites of atorvastatin due to impaired efflux transporter function. It would be interesting to understand better the pharmacokinetics profile of atorvastatin in these patients to confirm this hypothesis.

Non-genetic risk factors associated with SAMS include a personal or family history of muscle disease, female sex, older age, use of concomitant medications, a family history of CVD, obesity, hypertension, untreated hypothyroidism, liver or kidney disease, diabetes mellitus, history of smoking, alcohol abuse, type and high dose of statins^32,33^. To investigate the association between these covariates and SAMS, we first performed a simple logistic regression analysis, in which only family history of CVD showed a significant influence on SAMS. After performing a multivariable logistic regression analysis that considered only significant genetic factors adjusted for all environmental covariates (Table 5), we found that the effect of genetic variables on SAMS remained significant, even after adjustment. We also confirmed the role of family history of CVD on SAMS risk, which is consistent with available literature data^32,33^.

The strength of our study is that the cutoff value for CK was not set beyond the upper limit of the reference range, so all individuals with activity of CK above the reference range were considered cases. Another strength is that the influence of all non-genetic factors previously known in the literature that may contribute to the development of SAMS was excluded due to clearly defined exclusion criteria in the design of the study or the use of logistic regression analysis in the statistical processing of the results. The results obtained highlight the influence of the genetic factor in the pathogenesis of SAMS. The main limitations of this study are that no blood levels of atorvastatin were measured to confirm the association between pharmacokinetics and outcome and that the influence of genetic variants in other genes involved in the pharmacokinetics of atorvastatin, such as: *CYP3A4*, *SLCO1B1*, *SLCO2B1*, was not investigated, although they are targeted for the future research. However, we acknowledge the limitations of a single candidate gene analysis when studying most probably polygenic trait such as SAMS, but our goal was to conduct a hypothesis-driven focused research, taking into account the importance of the candidate gene in pharmacokinetics of atorvastatin, the functional implications of studied polymorphisms, but conflicting results of candidate gene association studies with SAMS. Another limitation is the subjective statement of patients regarding their muscle symptoms, whose impact was minimized by the high-quality design of the questionnaire. Also, it should be mentioned as a limitation that the study population was homogeneous in the terms of race, ethnicity, and ancestry. All patients belonged to the South Slavic ethnic group. It should be noted that the confidence intervals for significant *p* values in our study are wide. This could be explained to a large extent by the small sample size. However, it should be emphasized that although the sample in our study was small, the sample size and power sample calculations showed that the sample size was adequate for the given study design. We have demonstrated that the *ABCB1* gene plays an important role in the disposition of atorvastatin, but further prospective, multiple gene studies on a larger patient cohort are warranted. Future direction should also include a meta-analysis of this study’s findings along with previous studies, with an assessment of heterogeneity among this and the previous studies and possible publication bias, which would help provide the readers with a clearer answer regarding the true role of this candidate gene with SAMS.

We believe that *ABCB1* genotyping has a great potential to be incorporated into CPIC guidelines and clinical practice and that could enable early identification of patients at risk of development of SAMS, which would lead to improved statin therapy and better clinical management of patients.

## Materials and methods

### Study design and patient recruitment

A cross-sectional study was conducted at the Faculty of Medicine, University of Montenegro, from April 2021 to May 2022. Eligible patients were outpatients, who came for routine check-up, older than 18 years of age who were on atorvastatin therapy for at least a month and with baseline CK levels within the reference range. Subjects using drug inducers or inhibitors of transporter proteins and enzymes (OATP1B1, ABCB1, CYP3A4) involved in the metabolism of atorvastatin, using fibrates, abusing alcohol, having uncontrolled hypothyroidism, subjects diagnosed with renal or liver disease, psychotic, neurologic, oncologic, or autoimmune diseases, and having incomplete data were excluded from the study. Sample size was calculated according to the formula:

$$n={Z}^{2}\times \frac{p \times q}{{e}^{2}},$$

where n = sample size, Z = 1.96 for 95% confidence interval, *p* = prevalence of SAMS, q = 1-p, e = margin of error, 5%.

According to the literature data, prevalence of SAMS is 5–10% in the clinical setting^18^, so we calculated that the sample size should be in range of 73–138 subjects. We enrolled 110 participants. All included participants signed an informed consent form, and their demographic and clinical data were collected through medical records, and standardized questionnaires. The following data were collected: gender, age, existing comorbidities, family history of cardiovascular disease (CVD), body mass index (BMI), smoking status, alcohol consumption, physical activity, atorvastatin dose taken by the respondent, and duration of therapy. All variables were considered as covariates. Respondents indicated whether they have and/or had symptoms of SAMS, such as cramps, muscle pain, or muscle weakness, through a questionnaire based on the Naranjo Adverse Drug Reaction Probability Scale and the Statin-Associated Muscle Symptom Clinical Index^19,20^, and whether it resulted in them taking a lower dose of the drug, which was confirmed by medical record. Subsequently, study population was divided according to outcome into 2 groups: subjects who reported symptoms of SAMS and/or dose decrease, and/or had elevated CK levels outside the reference range were assigned to the atorvastatin-associated muscle symptoms (AAMS) group; whereas subjects who reported no SAMS symptoms, no dose decrease, and whose CK levels were within the reference range (0–170 IU/L for women, 0–190 IU/L for men) were assigned to the non-atorvastatin-associated muscle symptoms (N-AAMS) group. Patients with CK values outside the reference range in the medical report 3 months after atorvastatin initiation (information was found in medical record), were automatically included in the AAMS group. Patients with the CK values outside the reference range noted in the post-interview findings were placed in the AAMS group only if they also reported muscle cramps/pain by the questionnaire or if the atorvastatin dose was increased in the last 3 months.

### CK activity measurement and genotyping

A venous blood sample was collected from all subjects and placed in a tube containing clot activator and a gel separator, for CK activity measurement. CK activity was measured from serum according to the International Federation of Clinical Chemistry standardized method on the Beckman Coulter AU5800 analyzer the day after sample collection. A separate tube containing ethylenediaminetetraacetic acid (EDTA) was used for the collection of blood samples for genotyping. Whole blood samples were immediately stored at −80 °C until further analysis. Genomic DNA was isolated from the whole blood using the QIAamp DNA Blood Mini Kit (cat. no. 51106, Qiagen, Hilden, Germany), according to the manufacturer’s protocol. Quantification of the isolated DNA was performed using a UV/VIS spectrophotometer (Eppendorf BioSpectrometer® basic) and 10 ng DNA was used for further analysis. Genotyping of *ABCB1* polymorphisms was performed on the Bio-Rad CFX96 real-time PCR system, using the allele-specific real-time PCR method. The following TaqMan DME genotyping assays were used: rs1128503; assay ID: C___7586662_10, rs2032582; assay ID: C_11711720C_30 and C_11711720D_40, rs1045642 assay ID: C___7586657_20 (Thermo Fisher Scientific).

### Statistical analysis

Power sample size calculations were performed by G*Power (version 3.1.9.7, Heinrich Heine Universität Düsseldorf, Germany) to estimate the number of individuals per group for this study. According to literature data ^11,12^ we assumed 28% difference in proportion of TT genotype between AAMS and N-AAMS groups (0.38 vs. 0.10). Taking a power of 0.80 and a type 1 error rate of 0.05 we calculated a sample size of 40 patients per group. All analyzes were performed using statistical software GraphPad Prism 9.3.1 (GraphPad Software, San Diego, CA, USA). Categorical variables were presented as numbers and percentages, while continuous variables were expressed as mean ± standard deviation. Continuous variables were first tested for normality of distribution by D’Agostino-Pearson and Shapiro–Wilk tests and analyzed with the t-test or appropriate non-parametric test. χ-square or Fisher’s exact test were used for categorical variables. The distribution of genotypes was assessed for deviation from the Hardy–Weinberg equilibrium by use of the χ-square goodness-of-fit test. For haplotype phasing estimation and frequency calculations we used Arlequin software Version 3.11 (http://cmpg.unibe.ch/software/arlequin3/). Fisher’s exact test was used for association analysis between alleles, genotypes, haplotypes, diplotypes and SAMS. We performed multiple hypothesis tests Holme’s Step Down Procedure to adjust *p*-values for three different genotype models to account for multiple hypothesis testing. Simple logistic regression analysis was performed to assess the potential confounding influence of each covariate on the association with SAMS. After that, multivariable logistic regression analysis was performed and included only significant genetic factors adjusted for all environmental covariates. P-value less than 0.05 was considered to be statistically significant.

### Ethical approval

The study was conducted in accordance with the Declaration of Helsinki and the protocol was approved by the Ethics Committee of the University of Montenegro, Faculty of Medicine (No. 2050/7) in December 2020. Written informed consents were obtained from all participants.

## Data Availability

The datasets analyzed during the study are available from the corresponding author on reasonable request.
